# Performance of Plant-Produced Asphalt Containing Cellular Capsules

**DOI:** 10.3390/ma15238404

**Published:** 2022-11-25

**Authors:** Laura Traseira-Piñeiro, Tony Parry, Frank Haughey, Alvaro Garcia-Hernandez

**Affiliations:** 1 Nottingham Transportation Engineering Centre, Faculty of Engineering, University of Nottingham, Nottingham NG7 2RD, UK; 2 Atkins Ltd., 2 Chamberlain Square, Birmingham B3 3AX, UK; 3 Tarmac Trading Ltd., Station Approach, Essex CM20 2EL, UK

**Keywords:** stone mastic asphalt, ravelling, rutting, macrotexture, encapsulated rejuvenators, polymeric capsules, cellular materials, energy-absorption

## Abstract

This paper aims to assess the influence of encapsulated rejuvenators on plant-produced asphalt’s performance. The polymeric capsules are evaluated as cellular materials that deform and absorb energy while they experience a progressive collapse of their porous structure, rather than a simply means to release the rejuvenator. Additionally, variables during asphalt manufacturing that may affect their plastic deformation under loading are assessed too. Firstly, plant-produced asphalt’s mechanical and morphological properties were evaluated, including the capsules’ distribution and integrity after mixing. Then, results were contrasted with lab-produced asphalt under controlled conditions. Lastly, the capsules’ deformation was qualitatively evaluated using a FE model to verify findings from the testing campaign. It was concluded that (i) cellular capsules can resist mixing at an asphalt plant without compromising their performance; (ii) the deformation of the capsules affected asphalt’s stability by up to 13%, reduced the particle loss by up to 25% and increased asphalt’s macrotexture by 10%; (iii) to maximize their energy absorption, the cellular capsules must be part of the aggregate skeleton.

## 1. Introduction

Due to its high performance, Stone Mastic Asphalt (SMA) is one of the most popular surfacing materials in the UK, particularly on pavements with heavy traffic and heavy loads. The stone-on-stone contact between the coarse aggregates creates a strong skeleton, providing stability, whereas the voids are partially filled with a bituminous mortar composed of filler and fines, increasing durability.

The selection of the type and content of the binder can directly affect the mixture’s rutting resistance [1,2,3]. This wearing course asphalt can be manufactured in either batch-mix or drum-mix asphalt plants, with the latter being more cost-effective for large-scale production. Nevertheless, SMA’s performance is also influenced by the mixing period, the temperature profile (up to 180 °C) and the operation of the rotating drum (about 8 rpm) [4,5].

Asphalt cracking and ravelling are among the most prevalent distresses on the network. However, surface cracking on gap-graded mixtures is most likely to be caused by reflective cracking from the underneath layer, which constitutes a structural problem [6,7,8]. Ravelling, on the other hand, has a high occurrence on thin overlays and high-textured materials. The loss of aggregates, caused by a weak bond in the stone-on-stone contact, can be prompted either by cohesive failure of the binder or adhesive failure in the bitumen-aggregate interface. Although several factors may influence ravelling, such as bitumen ageing, it is a particularly concerning distress during wet seasons due to moisture that affects bitumen’s cohesive strength and adhesive bond in the interface region. Once initiated, asphalt ravelling rapidly intensifies in the surrounding areas, reducing pavement’s service life and requiring maintenance [9,10,11,12].

Rutting is also a concerning material distress, but SMA mixtures are known for its high stability against plastic shear deformations. In most cases, rutting occurs because the asphalt layer is not strong enough to bear the traffic loads, due to an inappropriate interlocking or aggregate distribution in the mixture. Consequently, rutting can accelerate pavement deterioration by reducing the drainage capacity and increasing the risk of moisture damage and bleeding [1,8].

Encapsulated rejuvenators were originally designed for enhancing asphalt self-healing by the progressive release of a rejuvenator that restores the properties of aged bitumen, promoting crack healing [13,14,15,16,17]. This mechanism requires: (i) mechanical loading to activate the capsules, (ii) a chemical interaction to restore the binder’s composition and, (iii) binder flow in a porous media, to seal the microcracks with the restored binder [18]. Therefore, researchers have been focused on evaluating the release and diffusion of the rejuvenator and estimating asphalt self-healing [13,14,15,16,17,18]. Although this mechanism is reliant on the test temperatures and resting times, the mechanical properties of these capsules were not affected by temperatures ranged between −20 °C and 100 °C [19]. Thus, their design and optimization were only constrained by the yield load required for resisting the forces and temperatures in the mixing and compaction stages at a laboratory scale, which was estimated to be approximately 10 N [20].

These capsules were later found to be more than just a release mechanism [21], as the polymer they are made of has a cellular structure with an elastoplastic behavior, characterized by a progressive collapse of the internal porous structure and subsequent absorption of energy under loading [22]. Although recent studies related asphalt’s increase of energy absorption on mixtures containing polymeric capsules with the reduction of the aggregate loss [21], not only the mechanisms underlying their deformation are still unclear, but also how the subsequent local re-compaction of the aggregates can affect asphalt’s performance.

This paper aims to assess the influence of polymeric capsules on plant-produced asphalt performance. These capsules are being examined as a cellular material that deform and absorb energy. Firstly, asphalt containing capsules was produced at an asphalt plant to evaluate the capsules’ distribution and integrity after mixing using industrial processes. Thereafter, to determine whether the aggregate skeleton that characterizes SMA mixtures was affected by the deformation of the capsules, asphalt’s stability, ravelling resistance, macrotexture, and void content were evaluated. The results were then corroborated lab-produced asphalt under controlled conditions, eliminating disparities on asphalt’s performance caused by the large-scale production. Lastly, FE modelling was used to verify findings from the testing campaign.

## 2. Materials and Methods

### 2.1. Polymeric Capsules and Manufacturing Process

A total of 23 kg of cellular capsules were required for this study. The materials employed to obtain the crosslinked calcium-alginate polymer that comprises the capsules’ cellular structure were sodium alginate (C_6_H_7_O_6_Na, bulk density: 0.7 g/cm^3^, flash point: 200 °C) and calcium chloride (CaCl_2_ 93% purity, relative density: 2.1 g/cm^3^, melting point: 770 °C). The cavities formed by the external ionotropic gelation technique contained a bitumen-compatible additive, used as a rejuvenator in prior research (sunflower oil, relative density: 0.92 g/cm^3^, smoke point: 230 °C) [13,14,15,16,17].

**Figure 1:**
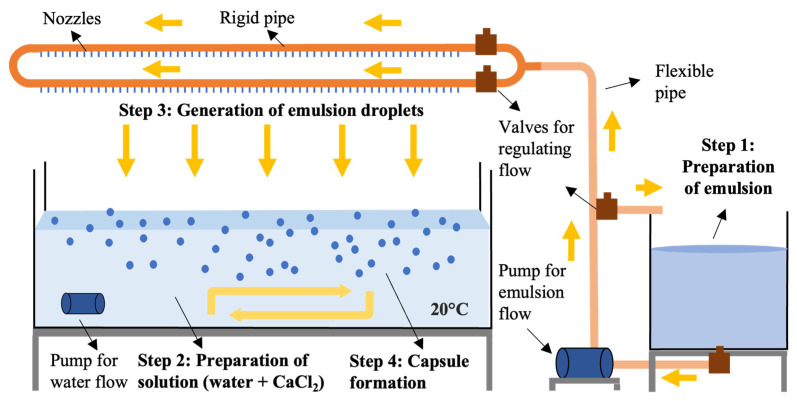
Diagram of the upscaled encapsulation procedure.

This study required upscaling the process to attain the required volume of capsules, see Figure 1, which comprised the same stages as the abovementioned literature [13,14,15,16,17]: (i) Preparation of a stable emulsion of water, sunflower oil and alginate using a high shear mixer (ratio o/w 0.12, a/o 0.18), (ii) preparation of a solution of water and calcium chloride (concentration of 2%), (iii) generation of emulsion droplets from a dropping system controlled by a pump, (iv) capsule formation from the droplets that fell into the calcium chloride solution and, (v) particle collecting and drying for 24 h at 50 °C. The final step required coating the capsules with limestone filler (<63 μm) to aid drying. After drying, the capsules were sieved to remove any excess filler, see Table 1, packed in melting bags of 5 kg and stored in a temperature-controlled room at 5 °C. Cubes of the same composition and approximate dimensions of 20 × 20 × 20 mm^3^ were also produced with an analogous manufacturing technique that did not require the drop formation step.

### 2.2. Asphalt and Specimen Preparation

Stone Mastic Asphalt, SMA 10 surf 40/60 (BS EN 13108-5 [23]), was produced at both Mountsorrel Tarmac Asphalt Plant (Loughborough, UK) and Nottingham Transportation Engineering Centre (NTEC) laboratory (Nottingham, UK). The materials used were graded granite aggregates (2.8 g/cm^3^), 40/60 pen bitumen (1.027 g/cm^3^, penetration at 25 °C of 52 × 10^−1^ mm, softening point of 51.3 °C), pellets of cellulose fibres (0.095 g/cm^3^) and cellular capsules (1.074 g/cm^3^).

At the Mountsorrel plant, asphalt was produced using a drum-mix facility, with a total mixing period of 27 s at 180 °C. The production was divided into four batches of 0.5 tons each. Asphalt gradation by weight was: size 14 mm, 5%; size 10 mm, 48.4%; size 6 mm, 16%; dust, 15.4%; filler, 8.5%; bitumen, 6.4%; fibres, 0.3%. The first batch (M1) had no cellular capsules, whereas the subsequent batches contained 0.5% of capsules by total weight. As no prior research has used industrial facilities to produce asphalt containing encapsulated rejuvenators, they were incorporated at different times of the mixing stage: with the aggregates in the second batch (M2); at second 5/27 of mixing in the third batch (M3), to evaluate if the maximum mixing time could damage the capsules; and at second 20/27 in the fourth batch (M4), to evaluate if the minimum mixing time could affect the distribution of the capsules; see Table 2. A total of 800 kg of loose asphalt was packed into boxes of approximately 10 kg and transported to the NTEC laboratory for asphalt specimen preparation. Additionally, the composition of the four batches was analyzed following BS EN 12697-1 (Annex B.3.1) [24], using methylene chloride as solvent, and BS EN 12697-2 [25].

At the NTEC laboratory, an analogous SMA mixture was produced under controlled conditions with 0% and 0.5% of cellular capsule content, to validate results obtained from testing plant-produced asphalt. The times for adding the cellular capsules during asphalt mixing are presented in Table 2, and the mixture’s gradation in Table 3. As the binder dosing in the plant is prone to undergo certain variation, four additional SMA mixtures were manufactured with the following binder contents: 4.5%, 5%, 5.5%, and 6.5%. Cellular capsules were added in the last 30 s of mixing when modifying the bitumen content, based on previous research at a laboratory scale [17]. The SMA mixtures required preheating aggregates and bitumen at 165 °C for eight and four hours, respectively, and a total mixing time of 3 min at 135 rpm.

SMA mixtures from the Mountsorrel plant were preheated at 170 °C for four hours before being transferred to metallic molds of 305 × 305 mm^2^ and compacted with a roller slab compactor to achieve the target air void content (3%). SMA mixtures from NTEC were directly transferred to the molds after mixing for compaction under the same conditions. The slabs’ target height for rutting testing was 50 mm, whereas 100 mm diameter cores for semi-circular bending and particle loss testing were extracted from 45 mm and 60 mm slabs. Semi-circular cores required trimming the top and bottom surfaces of the specimen, and adjusting the thickness to 40 mm. A notch of 4 × 8 mm^2^ was also made at the mid-point of the semi-core base. Lastly, cylinders of 35 mm diameter were cored from the slabs for CT-scanning.

### 2.3. Determination of Test Specimens’ Air Void Content

The air void content (AVC) was determined using Equation (1) from BS EN 12697-8 [26]. The asphalt mixtures’ maximum density ($\rho_{m})$ was calculated through procedure C from BS EN 12697-5 [27], and the specimens’ bulk density ($\rho_{b}$) was calculated following procedure C from BS EN 12697-6 [28].

(1) $$\mathrm{AVC}\;\left(\%\right)=\frac{\rho_{m}-\rho_{b}}{\rho_{m}}100$$

### 2.4. Determination of the Cellular Capsules’ Density

The density of the capsules’ after drying and sieving was analyzed using a gas displacement pycnometer (Micromeritics AccuPyc II 1340, Gloucestershire, UK) at room temperature (20 °C), testing a sample size of approximately 2 g. The density (g/cm^3^) was then calculated as the average of five measurements.

### 2.5. Imaging Techniques

The cellular capsules’ morphology was evaluated using the ImageJ software (Version 2.3.0). A total of 50 particles in their resting position were analyzed using plan-view images. The Equivalent Circular Diameter (*ECD*), circularity, and Aspect Ratio (*AR*) were calculated through Equations (2)–(4), respectively, based on the area of the capsules and the major and minor axis of the fitted ellipse The cellular capsules’ volume was estimated as the volume of the equivalent ellipsoid, using front-view images to determine the length of the third axis.

(2) $$Equivalent\;Circular\;Diameter=2\sqrt{Area/\pi }$$

(3) $$Circularity=\frac{4\pi Area}{Perimeter^{2}}$$

(4) $$Aspect\;Ratio=\frac{Major\;axis}{Minor\;axis}$$

The inner and outer structures of the capsules were documented using an FEI Quanta 600 Scanning Electron Microscope (FEI Company, Hillsboro, OR, USA). Beam settings were adjusted to a high accelerating voltage of 2 kV and spot size 3. Sample preparation for SEM analysis required: (i) Razor-cutting samples to expose the inner structure, (ii) individual particle embedding into an epoxy resin using a vacuum system to eliminate air, (iii) specimen polishing, (iv) plasma cleaning to remove any organic contamination from the sample and, (v) specimen platinum sputter coating for high-resolution imaging in the SEM microscopy.

Further analysis of the capsules’ inner structure required X-ray Computed Tomography scanning (CT scans) to characterize the cavities in which the bitumen-compatible additive is located. The equipment used was a Phoenix Nanotrom S (Waygate Technologies, Wunstorf, Germany), operating at 180 kV and 1.25 µm of spatial resolution, and the scans were processed on VGStudio MAX (Version 2.2.6) and ImageJ. CT scanning was also used to evaluate the capsule distribution and morphology on asphalt manufactured at the Mountsorrel plant, with a Phoenix Vtomex M (Waygate Technologies, Wunstorf, Germany), operating at 200 kV and 68 µm of spatial resolution. Images obtained from CT scanning were reconstructed with VGStudio MAX and ImageJ. As the volume of loose capsules presented a normal distribution, undamaged capsules were identified in the CT-scans as those whose volume was within the estimated average volume ± 2 standard deviations [29]. Lastly, the total percentage of undamaged capsules was calculated as the ratio between the volume of undamaged capsules to the total volume of the capsules in the mixture

### 2.6. Uniaxial Compression Test

Compression testing was performed on 50 cellular capsules using an Instron 5969 (Wycombe, UK) at 20 °C and 50% humidity, 0.3 mm/min displacement rate. An optical extensometer was coupled to the equipment to obtain accurate readings of the contact area between the steel plate and the capsule to determine the corresponding stress–strain curve. Due to the size and morphology limitations of the capsules, 10 cubic samples made of the same polymer were tested to estimate the Poisson modulus (υ). An analogous procedure was followed to test lab-produced asphalt samples with approximate dimensions of 90 × 90 × 50 mm^3^ on an Instron 5985 with a 10 mm/min displacement rate.

### 2.7. Semi-Circular Bending Test

The semi-circular bending test was used for quality control of asphalt from the Mountsorrel plant [30]. The equipment used was an Instron 1332, operating at a 5 mm/min. Samples were preconditioned at −10 °C for 24 h. The test setup entailed a pair of 20 mm diameter rollers with a span of 80 mm to support the sample base and a plate of 8 mm thickness to load the top of the semi-cores. Calculations of the maximum stress at failure ($\sigma_{max}$) and the fracture toughness (K) followed BS EN 12697-44 [31], using Equation (5) and Equation (6), respectively. $F_{max}$ is the maximum force, D and t are the diameter and thickness of the sample, $I_{1}$ is the normalized mode I stress intensity factor, and a is the notch depth of the specimen. A total of 10 samples were tested for each batch of asphalt.

(5) $$\sigma_{max}=F_{max}/\left(Dt\right)$$

(6) $$K=\sigma_{max}I_{1}\sqrt{\mu a}$$

The modified Z-score ($MZ_{i}$), which uses the Median Absolute Deviation (MAD) as an estimator, was calculated to identify and discard outliers resulting from random variations during testing, see Equation (7), where $\tilde{x}$ represents the median value of the dataset and $x_{i}$ represents a single value. According to [32], results greater than 3.5 were removed from the dataset as outliers.

(7) $$MZ_{i}=\frac{0.6745\left(x_{i}-\tilde{x}\right)}{median_{i}\left\{\left|x_{i}-\tilde{x}\right|\right\}}$$

### 2.8. Aggregate Loss Resistance Test

Asphalt ravelling resistance was assessed with the Cantabro test, following BS EN 12697-17 [33], to evaluate if asphalt’s particle loss could be affected by the capsules and their deformation under impact loading. Sample sets of five specimens were individually tested in the Los Angeles drum without steel balls, rotating at 31 rpm for 300 cycles. The samples’ mass was recorded before and after the test, and the Particle Loss (*PL*) was calculated through Equation (8), where $W_{i}$ is the specimen’s mass before testing and $W_{f}$ the mass of the sample after 300 cycles. Lastly, this value was expressed as the average of the sample set. After testing, the particles lost from the cores were sieved to obtain their gradation.

(8) $$PL\;\left(\%\right)=100\frac{W_{i}-W_{f}}{W_{i}}$$

### 2.9. Quantification of Additive Released

The volume of bitumen-compatible additive released by the cellular capsules after the Cantabro test was calculated through Fourier Transform Infrared Spectroscopy (FTIR) [16,17]. The equipment used was a Bruker Optics IFS66 spectrometer (Karlsruhe, Germany) with an ATR attachment, operated in the absorption mode in the wavenumber range of 400 to 4000 cm^−1^ with 4 cm^−1^ of resolution. Sample preparation required: (i) heating tested asphalt samples for 20 min at 160 °C, (ii) extracting bitumen samples, (iii) homogenizing the samples. To determine reference values, virgin bitumen was blended with sunflower oil.

The absorption spectra were normalized in the range of 1720 to 1770 cm^−1^, as seed oils can be identified due to an intensification at approximately 1745 cm^−1^ (C=O stretching vibration). The absorbance area between curves with and without capsules was calculated using numerical integration. Then, the percentage of sunflower oil released was determined by Equation (9), where Abs.area cellular capsules is the absorbance area of bitumen from tested samples containing capsules, and Abs.area oil is the reference absorbance area of virgin bitumen blended with sunflower oil. An analogous procedure was followed to identify traces of the calcium alginate polymer after asphalt manufacturing with a distinct peak at 3320 cm^−1^ (−OH stretching vibration) [34].

(9) $$Oil\;released\;\left(\%\right)=\frac{Abs.area\;capsules}{Abs.area\;oil}$$

### 2.10. Rutting Resistance Test

The mixture’s stability was studied with the Hamburg wheel tracker (Cooper, Ripley, UK) under BS EN 12697-22 [35], to evaluate if asphalt’s permanent deformation could be affected by the capsules and their deformation under cyclic loading. Samples were tested fully submerged in water at 45 ± 1 °C after being preconditioned for 4 h. The test stopped after tracking the samples at 26.6 rpm under a 705 ± 4.5 N load for 10,000 cycles. Measurements of the rut depth were recorded through procedure B from the standards [35], in 100 points equally spaced. The proportional rut depth (PRD) was calculated as Equation (10), where $d_{n}$ is the vertical displacement after n load cycles in millimeters, $d_{0}$ is initial vertical displacement (in mm), and h is the specimen thickness (in mm).

(10) $$\mathrm{PRD}\;\left(\%\right)=100\frac{d_{n}-d_{0}}{h}$$

A laser texture scanner (Ames model 9400, Ames, IA, USA) was used to evaluate whether asphalt’s macrotexture was affected by the capsules’ deformation after wheel tracking. Regions of 100 mm × 70 mm were analysed on asphalt slabs before and after being tested in the Hamburg Wheel tracker, scanning 150 segments in each region to calculate the Mean Profile Depth (MPD). The generated data were filtered using a 3 mm Butterworth second-order filter before processing. The data was collected and processed under BS EN ISO 13473-1 [36].

## 3. Finite Element Modeling

Previous studies evaluated the capsules as cellular materials that deform under loading [21], experiencing a progressive collapse of their porous structure that allowed them to absorb energy [22]. In addition, the experimental campaign of this paper revealed that their performance may be affected by the binder dosing. Hence, to corroborate findings from experimental testing, Abaqus (Version 2022) was used to model the deformation of a capsule between two stones when applying uniaxial compression, studying five scenarios in which the area of mortar surrounding the stone-capsule-stone matrix was progressively increased. Then, a qualitative study was made by comparing the results from the simulations.

### 3.1. Microstructural Model

A two-dimensional model was constructed based on a simplified case study of a circular capsule (1.6 mm diameter) between two stones subjected to a uniaxial compression, variating the area of mortar surrounding the stone-capsule-stone. This structure was enclosed by rigid body parts revested with a layer of asphalt. Boundary conditions were applied to the base of the model, limiting all displacements and rotations, and to the top plate, imposing a vertical displacement of 0.35 mm to apply compression. A limitation of this approach is the assumption of plain stress in 2D models. Additionally, the selected geometries for the microstructural model have not been generated from asphalt scans, hence, results are only qualitative in nature.

In the interface between the solid skeleton and asphalt mortar, a surface-based cohesive behavior with a traction-separation law was defined using the default contact enforcement method and friction (coefficient of 0.4) [37]. Friction was also defined between the rigid body boundaries of the model and the corresponding layer of asphalt that revested them (coefficient of 0.6) [38]. Quadratic triangular elements were used to discretize the model.

### 3.2. Determination of Materials’ Properties

Granite aggregates are assumed linear elastic, asphalt mortar is assumed linear viscoelastic, and the capsules are assumed to be elastoplastic. Table 4 shows the input parameters for the material properties.

**Figure 2:**
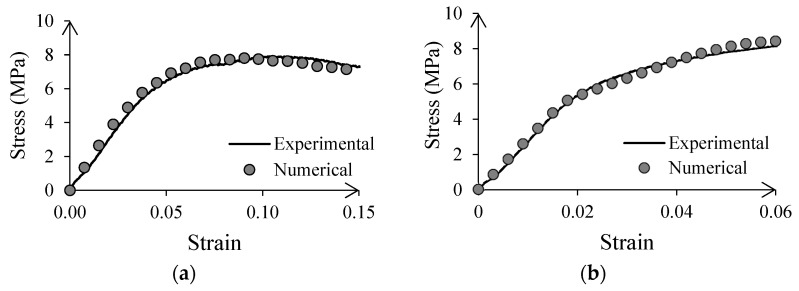
Experimental and numerical results of uniaxial compression testing of: (**a**) Cellular capsules and, (**b**) stone mastic asphalt.

The elastoplastic properties of the capsules were determined from the stress–strain curves obtained during uniaxial compression of the material, including the Young modulus (E) and the plastic deformation and stresses after yielding [39]. Then, they were verified by simulating uniaxial compression testing, and comparing the predicted behavior with the experimental data, see Figure 2a. An analogous procedure was used to determine the properties of the asphalt layer that revested the model’s rigid body boundaries (see Figure 2b) before the initiation and propagation of macrocracks when it reached a strain of 0.06.

Viscoelasticity in Abaqus is defined by Prony series expansion of the dimensionless shear relaxation modulus, based on the generalised Maxwell model [40,41]. It can be expressed in the frequency domain with Equations (11) and (12), in which $G^{\prime }\left(\omega \right)$ and $G^{\prime \prime }\left(\omega \right)$ are the storage and loss modulus at an angular frequency ω, $G_{0}$ is the instantaneous shear modulus and $t_{i}$ and $g_{i}$ are the dimensionless Prony series parameters, which can be determined from the calibration of frequency dependant data from the Dynamic Shear Rheometer (DSR).

(11) $$G^{\prime }\left(\omega \right)=G_{0}\left(1-\overset{n}{\underset{i=1}{\sum }}g_{i}\right)+G_{0}\left(\overset{n}{\underset{i=1}{\sum }}\frac{g_{i}t_{i}{}^{2}\omega^{2}}{1+t_{i}{}^{2}\omega^{2}}\right)$$

(12) $$G^{\prime \prime }\left(\omega \right)=G_{0}\left(\overset{n}{\underset{i=1}{\sum }}\frac{g_{i}t_{i}\omega }{1+t_{i}{}^{2}\omega^{2}}\right)$$

Consequently, frequency sweep tests were conducted on asphalt mortar (bitumen and aggregates up to 1 mm) at various temperatures (5 °C to 75 °C). Then, a master curve was constructed at a reference temperature of 20 °C using the WLF shift factors from the time superposition principle (see Equation (13)) combined with a sigmoidal fit function (see Equation (14)). ω and $\omega_{r}$ are the test and reduced frequencies, T and $T_{r}$ the test and reference temperatures, $C_{1}$, $C_{2}$, δ, λ, κ, γ are the fitting parameters, and $G^{*}$ and ϕ are the complex shear modulus and phase angle obtained from the test.

(13) $$log\left(\omega_{r}\right)=log\left(\omega \right)-\frac{C_{1}\left(T_{r}-T\right)}{C_{2}\left(T_{r}-T\right)}$$

(14) $$log(G^{*})=\delta +\frac{\lambda }{1+e^{\left(\kappa +\gamma log\left(\omega_{r}\right)\right)}}$$

(15) $$G^{\prime }=\left|G^{*}\right|\cos\phi ;\;G^{\prime \prime }=\left|G^{*}\right|\sin\phi$$

(16) $$G_{0}=\frac{E_{0}}{\left(1+\nu \right)}$$

**Figure 3:**
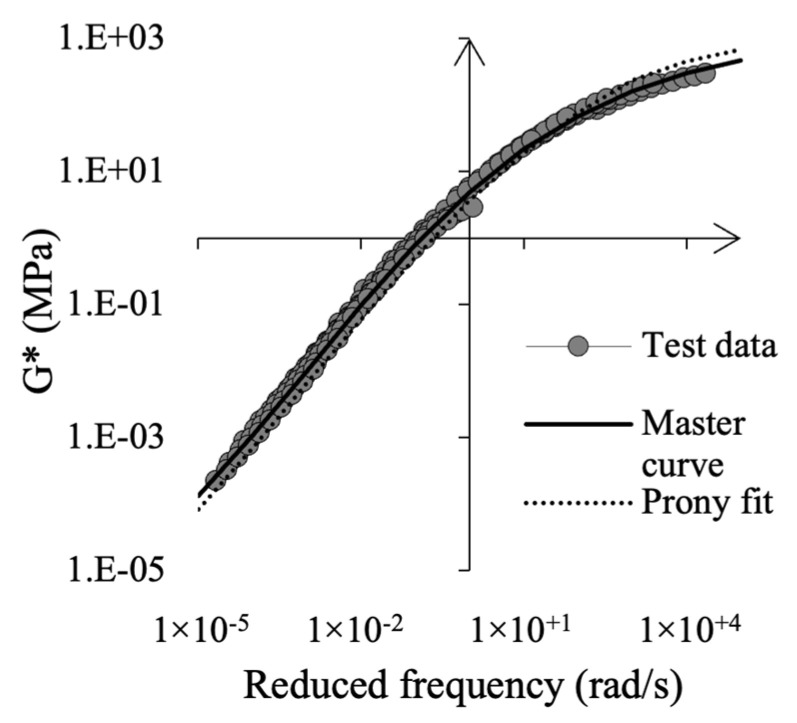
Results from DSR of asphalt mortar, master curve fitting and Prony series fitting at 20 °C.

Then, the Prony series parameters (see Equation (15)) were calibrated to asphalt mortar master curves of $G^{\prime }\left(\omega \right)$ and $G^{\prime \prime }\left(\omega \right)$, using the solver from MCalibration, see Figure 3. Lastly, viscosity needs to be defined along with elasticity in Abaqus, and the $E_{0}$ modulus was obtained from Equation (16), where ν is the Poisson ratio and $G_{0}$ the instantaneous shear modulus.

### 3.3. Evaluation of Results from the Simulation

The deformation of the capsule after applying compression was evaluated by the aspect ratio (AR), see Equation (4), which expresses the ratio between the major and minor axes of the fitted ellipse. A value of 1 describes a circle, whereas an increasing aspect ratio depicts a greater difference between both axes, hence, higher deformation of the capsule and higher energy absorbed due to the gradual collapse of its internal cellular structure [22].

Both the capsule’s aspect ratio and stiffness of the model were calculated in the five simulations. However, to determine if the cellular capsules may have also contributed to the stiffness of the model, these five simulations were repeated, but assigning granite properties to the circular cellular capsule. Then, the stiffness of the stone-capsule-stone model was divided by the stiffness of the stone-stone-stone model for each case and named as relative stiffness.

## 4. Results and Discussion

### 4.1. Polymeric Capsules Physical and Mechanical Properties

The cellular capsules were characterized to determine the impact of the upscaled manufacturing methodologies on their physical and mechanical properties. The median diameter of the capsules was 1.6 mm (Q2), and 50% of the ordered data set ranged between 1.4 mm (Q1) and 1.9 mm (Q3), as in [16]. The capsules presented an elongated shape, with a notably high median aspect ratio of 1.45 and circularity values below 1 (median of 0.88). The median volume of the equivalent ellipsoid was 1.7 mm^3^.

**Figure 4:**
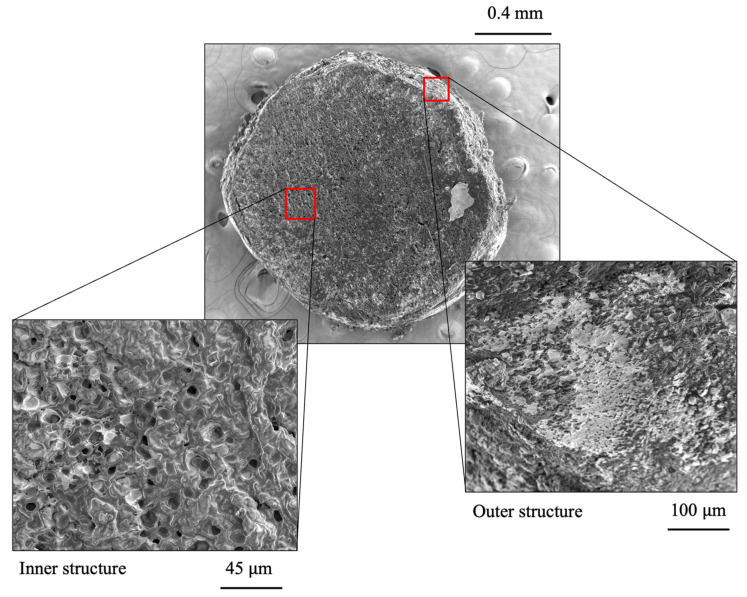
SEM images of the inner and outer structure of a bisected cellular capsule.

Figure 4 presents detailed views of the capsules’ inner and outer structure. The external structure did not present any pores, whereas a porous matrix constituted the internal one with closed cells generated from cross-linking sodium alginate with calcium ions. The cellular capsules comprised 47.1% calcium alginate, 46.7% additive, and 6.2% air. The bitumen-compatible additive used as a rejuvenator in prior research [13,14,15,16,17] was enclosed in cavities of 4.9 ± 4.8 μm diameter (average ± standard deviation), while the confined air was in voids of about 3.3 ± 2.1 μm diameter.

Under compressive loading, the cellular capsules exhibited an elastoplastic behavior. The average yield load was 9.5 N, in accordance with findings from [20], and was dependent on the diameter of the capsules, as described by a linear regression model (y = 8.68d − 4.82) with residuals normally distributed and a coefficient of determination (R^2^) of 0.75, that reported a meaningful level of predicted accuracy [42]. After yielding, the capsules showed strain hardening due to the plastic deformation, up to 0.15 of strain. Lastly, rather than experiencing a brittle fracture, the internal microstructure showed in Figure 4 initiated a gradual collapse with the subsequent release of additive, allowing the capsule to keep absorbing energy. The average Poisson ratio was 0.34, a similar value to gels, dental composites, and rubber [43].

### 4.2. Polymeric Capsule Distribution and Integrity on Plant-Produced Asphalt Samples

**Figure 5:**
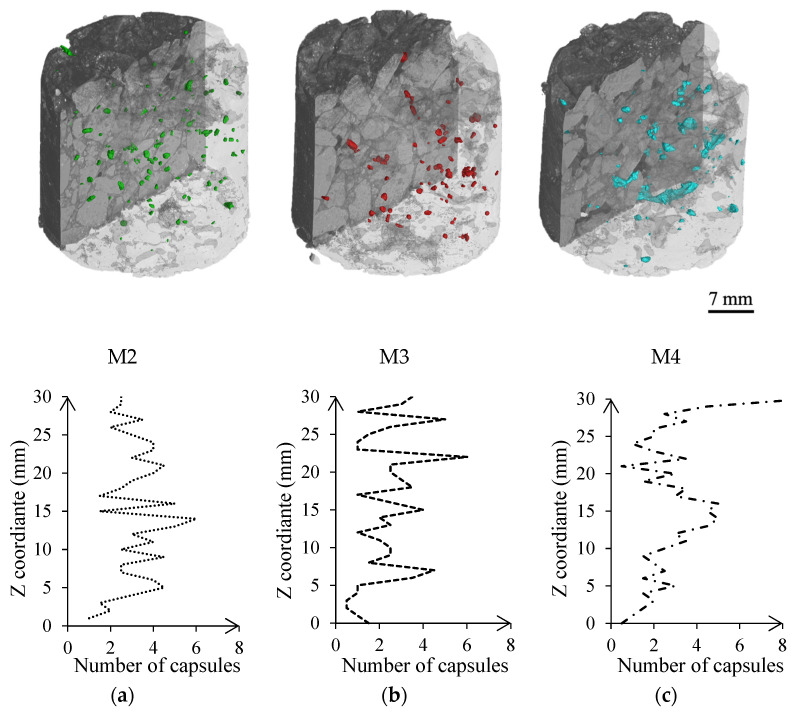
3D reconstruction of plant-produced asphalt specimens containing capsules, from batches: (**a**) M2, (**b**) M3, (**c**) M4.

Asphalt cores from plant-produced asphalt containing capsules were CT-scanned, see Figure 5, to evaluate if mixing at an asphalt plant could affect their distribution and integrity. Although their distribution throughout the asphalt was uniform, see Figure 5, some areas of the M4 batch had a higher concentration of capsules on the surface and center of the scanned cores, suggesting that the mixing drum took longer to distribute the capsules properly. Further analysis reported an average number of capsules (±2 standard deviation) per 3 mm of the height of 9 ± 4, 6 ± 4, 8 ± 6 in M2, M3, and M4, respectively. Undamaged capsules represented about 88 ± 3% of the total volume of capsules in M2, 85 ± 5% in M3, and 96 ± 1% in M4. Their morphology was not affected by the mixing process, as the capsules displayed similar median aspect ratio and circularity values as loose capsules (AR of 1.45, circularity of 0.92).

No more than 15% of the capsules were affected by the mixing process at an industrial scale. Moreover, adding the capsules at the end of the mixing resulted in less probability of damage, but also in a poorer distribution. Lastly, no traces of calcium alginate were identified in the FTIR analysis of the bitumen after mixing and compacting the asphalt, suggesting that the calcium alginate did not dissolve or intimately mix with bitumen.

### 4.3. Characterization of Plant-Produced Asphalt

The composition of plant-produced asphalt can be prone to a certain variability associated with the technologies employed to dry the aggregates and dose the materials. Therefore, prior to asphalt’s evaluation under impact loading (Cantabro test) and cyclic loading (Hamburg wheel-tracking test), plant-produced asphalt was subjected to a compositional analysis to detect any inconsistencies, and semi-circular testing was performed to assess whether disparities in asphalt composition might impact its performance.

**Figure 6:**
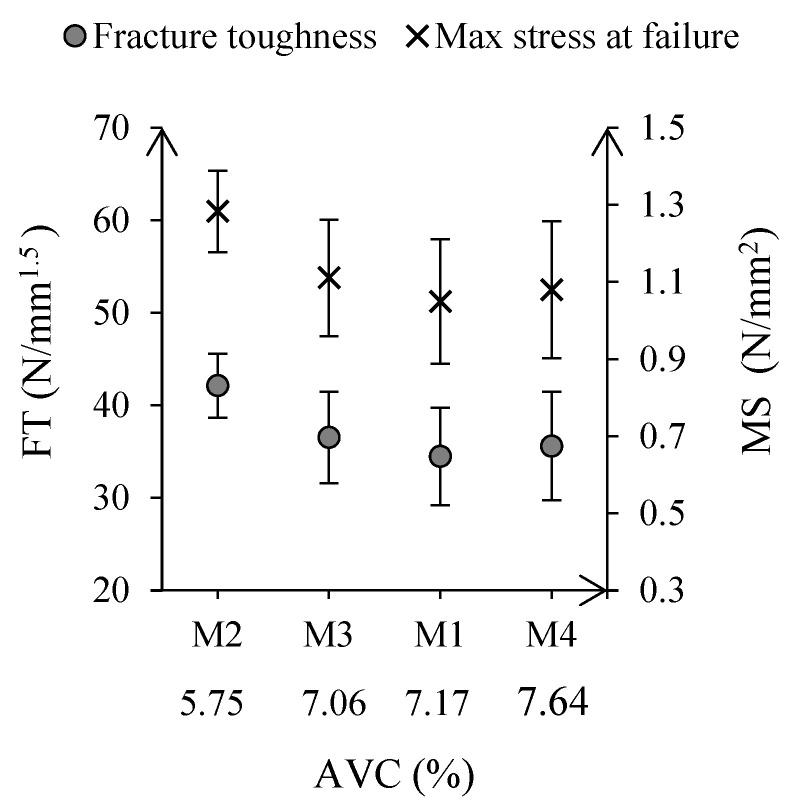
Results from semi-circular bending test and air void content calculations.

Based on Table 5, significant variations were found in the binder content, outside the 6.4% target by up to 2.3%. However, these variations may not have prevented a homogeneous distribution of the capsules during asphalt mixing, see Figure 5. According to semi-circular bending test results, see Figure 6, M2 exhibited the highest fracture toughness, while mixtures M1, M3, and M4 presented similar fracture resistance values. Furthermore, as reported by [44], asphalt resistance to cracking severely decreased in batches with a higher percentage of voids. No outliers were found in the data sets, and further statistical analysis (Student *t*-test) revealed that M2 void content and fracture toughness were significantly different from M1, M3, and M4 (*p*-value < 0.05). Hence, these results indicate that asphalt’s composition must be considered when evaluating the performance of the plant-produced asphalt batches.

### 4.4. Evaluation of Plant-Produced Asphalt Performance

Due to the capsules’ cellular nature and their ability to deform and absorb energy, there was no assessment of asphalt’s crack-healing. Instead, asphalt’s loss of stones, resistance to plastic deformations and macrotexture measurements were evaluated to determine if the aggregate skeleton that characterises SMA mixtures was affected by the deformation of the capsules under cyclic and impact loading.

**Figure 7:**
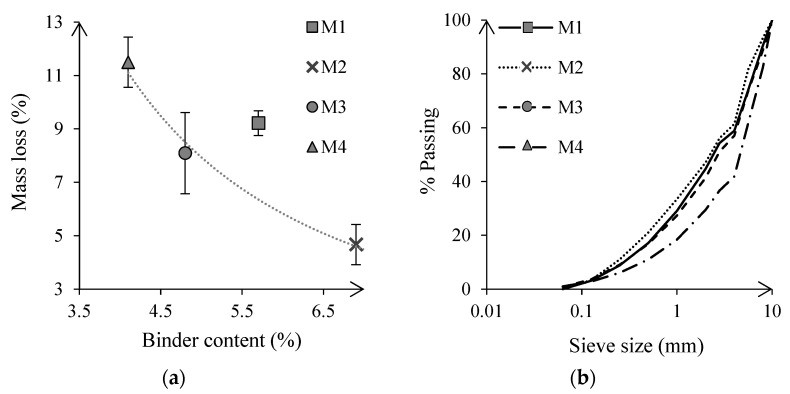
Results from the Cantabro test: (**a**) Percentage of mass loss and (**b**) aggregate gradation of the lost aggregates.

The Cantabro test showed a reduction of the aggregate loss in asphalt containing capsules of 49% and 12% in batches M2 and M3, as shown in Figure 7a, whereas it increased by 25% in the M4 batch. However, based on asphalt’s characterization from Section 4.3, these results were highly influenced by variations in the binder dosing, see Figure 7a, in which a power law was fit with a coefficient of determination of 0.98. Besides, the percentage of coarse aggregates lost increased with the mass loss, see Figure 7b.

Furthermore, FTIR analysis of the recovered binder revealed that, during testing, the capsules released about 66.8% and 62.5% of the additive in batches M4 and M3, and 48.0% in batch M2. These results suggest that the capsules experienced a plastic deformation that yielded in a release of additive during testing. Additionally, the high release additive in batch M4 could have been influenced not only by the binder dosing, but also by the mixing process, as the 3D reconstruction of batch M4 (Figure 5) showed some areas with a higher concentration of capsules, that might have facilitated their deformation and release of additive.

**Figure 8:**
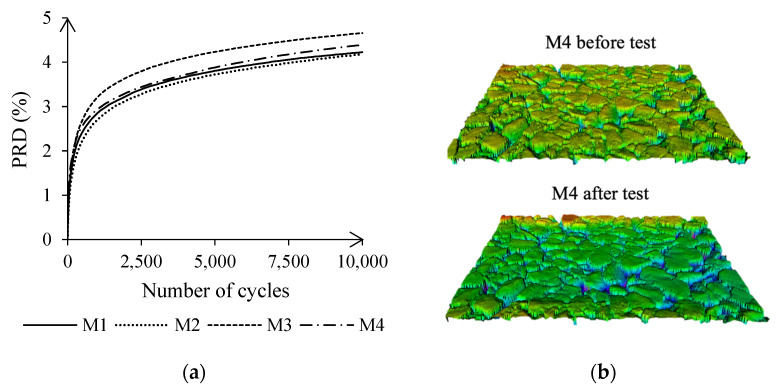
(**a**) Proportional rut depth of asphalt samples after wheel/tracking and (**b**) reconstruction from the scanned lines of a specimen in the mid-center before (MPD of 1.50 mm) and after (MPD of 1.56 mm) wheel tracking.

All four asphalt mixtures showed a proportional rut depth below 5%, or 2.55 mm after wheel-tracking, see Figure 8a. The highest plastic deformation was reached in asphalt batches M3 and M4, which contained capsules but also presented the lowest binder content. Therefore, differences on the binder’s dosing reported in Section 4.3 may have affected the deformation of the capsules under cyclic loading. Additionally, asphalt’s macrotexture measurements before testing showed higher MPD values on samples containing capsules: M1, 1.43 mm; M2, 1.45 mm; M3, 1.57 mm; M4, 1.50 mm. After testing, the MPD was increased by an average of 2% due to the wheel that, during testing, worn away the initial thick film of binder, see Figure 8b.

There is no clear indication of whether the industrial plant’s mixing stage affects the capsules’ ability to deform and absorb energy. Additionally, disparities on binder’s dosing affected asphalt’s performance, but their influence on the deformation of the capsules could not be quantified. Hence, a laboratory validation under controlled conditions is required to assess separately the effect of the capsules on asphalt performance when modifying: (i) the mixing stage in which they are incorporated, (ii) the binder content of the batch.

### 4.5. Validation of Results at the Laboratory Scale: Effect of Mixing Time

In light of the results from Section 4.4, an equivalent SMA mixture with 0% and 0.5% of cellular capsules was manufactured at lab-scale, to determine if the mixing time might have affected the capsules’ performance. The capsules were added at different stages of the mixing process, as defined in Table 2, and the test results from mechanical testing and morphological assessments are summarized in Table 6.

Although the volume of voids and the particle loss decreased on asphalt samples containing capsules, no evidence of a correlation between these parameters and variations in the mixing process was found. A student *t*-test revealed that samples containing 0% and 0.5% of capsules presented substantially different mass loss results after conducting the Cantabro test (*p*-value < 0.05). Besides, adding the capsules at 50% of the mixing process or before reduced the mass loss by an average of 38%, compared to an average reduction of 25% when added after 50% of the mixing. The proportional rut depth seemed to be reduced when adding capsules at about 50% and 75% of the mixing interval. An excessive mixing time might have released part of the additive, affecting the binder and hence also asphalt’s permanent deformation. However, mixing at the end of the process may have left some areas with a higher concentration of capsules, as seen in the plant-produced asphalt CT-scans (Figure 5), increasing rutting.

Lastly, asphalt macrotexture on samples without capsules had a steeper increase after wheel tracking, of about 9%, when compared to those containing capsules, whose MPD increased by an average of 3% but with no statistically significant differences when changing the mixing time of the capsules.

### 4.6. Validation of Results at the Laboratory Scale: Effect of Bitumen Content Variations

**Figure 9:**
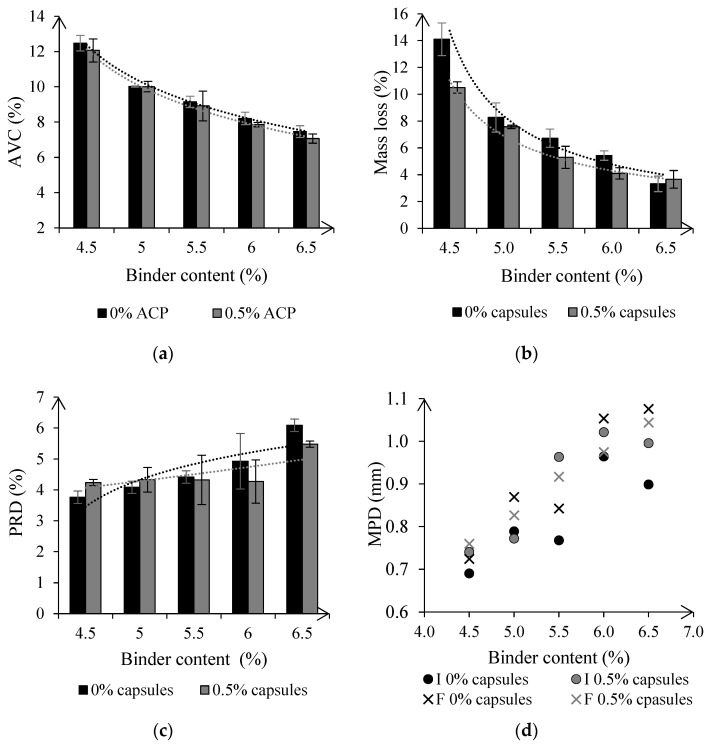
Testing of NTEC asphalt mixtures with different binder contents with 0% and 0.5% of cellular capsules: (**a**) AVC values of asphalt mixes, (**b**) ravelling resistance, (**c**) rutting resistance and (**d**) initial and final asphalt’s macrotexture during the Hamburg wheel tracking test.

Based on results from Section 4.4, an equivalent SMA mixture with 0% and 0.5% of cellular capsules was manufactured at lab-scale, to determine if variations in the binder dosing might have influenced the capsules’ deformation. Five asphalt mixtures were manufactured with different binder contents, and the test results are summarized in Figure 9.

Asphalt’s AVC was barely affected by the presence of capsules, and decreased by approximately 3% on samples with capsules regardless of the bitumen content, see Figure 9a, which is in line with results from Section 4.5, see Table 6. Moreover, Figure 9b shows that asphalt resistance to stone loss was highly dependent on the bitumen content, following a similar power law to that of Figure 7a. The capsules exhibited a greater performance when reducing the binder content of the asphalt mixture, decreasing the loss of stones by up to 25% compared to asphalt without capsules, see Figure 9b. Besides, this finding suggests that when asphalt’s binder content is increased, the cellular capsules’ deformation and subsequent energy absorption may be limited. This may stem from the capsules being suspended in the bitumen rather than constituting part of the aggregate skeleton.

Asphalt rutting increased with the bitumen content, see Figure 9c. However, this increase was gradual in samples containing 0.5% capsules, whereas the development of plastic deformations was steeper in asphalt without capsules. As a result, asphalt’s stability was improved by adding capsules for bitumen contents higher than 5%, matching the results obtained from the evaluation of plant-produced asphalt, see Figure 8, and the lab verification from Section 4.5, see Table 6. Under the test conditions, the capsules, which are prone to deformation, may act as a micro reinforcement of the mastic if there is a high bitumen content, improving the mixture’s stability.

Besides, asphalt macrotexture was also affected by the addition of cellular capsules, as shown in Figure 9d. Before wheel tracking, asphalt MPD values on samples containing capsules ranged between 0.75 mm and 1.05 mm, compared to 0.70 mm and 0.85 mm for asphalt without capsules. After testing, asphalt MPD on specimens with 0.5% of capsules fluctuated in the same range. In contrast, asphalt texture increased on samples without capsules, with an MPD varying from 0.77 mm to 1.09 mm, which was also observed in the lab verification from Section 4.5, see Table 6. According to [45], MPD values in asphalt with and without capsules are acceptable despite these variations. As asphalt’s macrotexture contributes to water evaporation on its surface [46], adding capsules will reduce the risk of aquaplaning in wet conditions.

### 4.7. Numerical Validation of Results Using FEM

Results from Section 4.4 and Section 4.6 showed that the performance of the capsules was influenced by variations in the binder content. As cellular materials, these capsules suffer a progressive collapse of their porous structure when deformed, absorbing energy [21]. Hence, a microstructural model was used for a qualitative analysis, in which five scenarios with a varying area of mortar surrounding a capsule in between two stones were evaluated.

**Figure 10:**
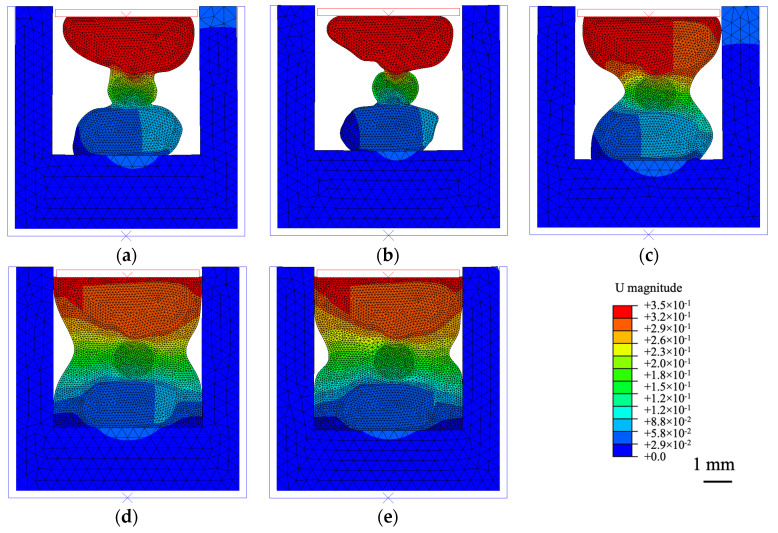
Total displacement U (in mm) in the FE model after applying uniaxial compression for the five variations in the case study altering the area of mortar. From (**a**–**e**), AR of the capsule after loading: 1.35, 1.30, 1.20, 1.14, 1.12.

The results of the simulations are presented in Figure 10. The model with the lowest volume of mortar, see Figure 10a, showed the highest deformation of the capsule, with an aspect ratio of 1.35. Then, as the area of mortar was gradually increased in the subsequent simulations, the deformation of the capsule was also reduced, as shown by the reduction of the aspect ratio up to a value of 1.12 in the last simulation, see Figure 10e. Therefore, this model could justify the test results from the particle loss in Figure 9b, in which the performance of the capsules increased as the binder content was reduced, increasing their deformation and energy absorption.

**Figure 11:**
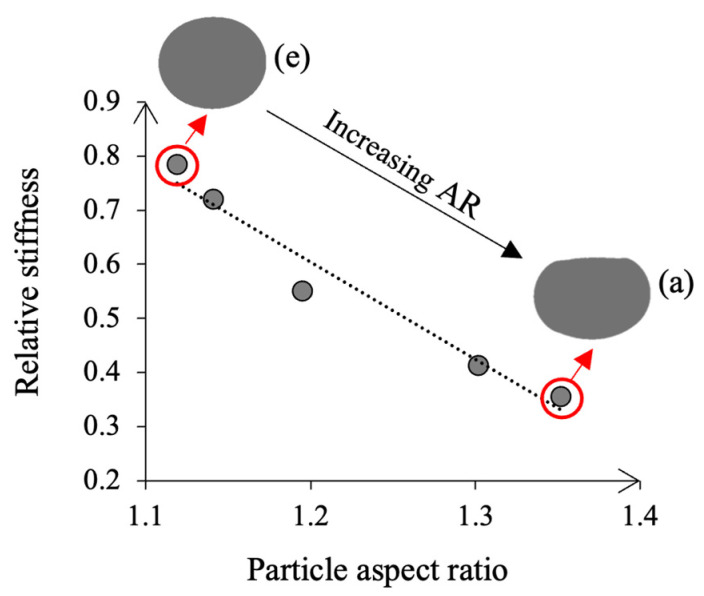
Correlation between the aspect ratio and the relative stiffness of the FE model and schematic representation of the capsules’ deformed shape in cases (a) and (e).

A growing area of mortar also increased the model’s stiffness, which ranged from 29.20 N/mm in simulation (a), to 51.36 N/mm in simulation (e). Besides, the capsules may have also contributed to this progressive rise in the material’s stiffness, as seen in Figure 11. If the capsules did not alter the model’s overall stiffness, the relative stiffness values of the simulations would remain constant for different deformations of the capsules. There was, however, a correlation between both parameters, described by linear regression (R^2^ of 0.96), see Figure 11. Therefore, this model could justify the test results from rutting testing in Figure 9c. A low binder content might increase the deformation of the capsule and hence, asphalt rutting. However, a high binder content might prevent the capsules from deforming, increasing the overall stiffness and therefore, reducing asphalt rutting.

## 5. Conclusions

The performance of plant-produced asphalt containing encapsulated rejuvenators was assessed evaluating the capsules as cellular materials that deform and absorb energy, rather than a simply means to release the rejuvenator. Firstly, the morphological and mechanical properties of asphalt mixtures produced at the asphalt plant were evaluated through experimental testing. The results were then contrasted with those obtained from testing at the laboratory scale under controlled conditions. Lastly, a simplified FE case study verified the findings from experimental testing with a numerical simulation. As a result, the following conclusions were drawn:

- The capsules can be incorporated into an asphalt plant without compromising their integrity or their distribution within the asphalt.
- The mixing stage in which the capsules were incorporated during asphalt manufacturing did not yield significant differences in lab-produced asphalt’s morphological and mechanical properties.
- The aggregate loss resistance increased on asphalt containing cellular capsules with the deformation of the capsules. Asphalt’s stability was also affected by the addition of capsules. Below a certain binder content, the capsules can deform and absorb energy, increasing rutting.
- Asphalt’s macrotexture increased on asphalt containing cellular capsules. In this asphalt with capsules, the MPD was barely affected by the Hamburg wheel tracking test. Asphalt without capsules showed a substantial increase in its macrotexture after Hamburg wheel tracking testing.
- The mixture’s binder content influences the deformation and energy absorption of the capsules. These need to constitute part of the stone-on-stone contact for maximizing their deformation. If they are floating in mortar, they will reduce their performance or remain inactive.

## Figures and Tables

**Table 1:** Polymeric capsules’ composition.

Composition	Weight (%)
Sunflower oil	67.0%
Limestone filler	15.4%
Sodium alginate	13.1%
Water	4.5%

**Table 2:** Times for adding cellular capsules into the asphalt during the mixing stage.

Mixing Achieved (%)		Mixing Time (min)	
Asphalt Plant	Laboratory	Asphalt Plant	Laboratory
0%	0%	00:00	00:00
19%	20%	00:05	00:36
-	50%	-	01:30
74%	75%	00:20	02:15
**Total mixing time**		00:27	03:00

**Table 3:** Composition of the NTEC laboratory asphalt mixture.

Sieve Size		Passing (%)
14	mm	100.0
10	mm	97.0
8	mm	84.3
6.3	mm	69.6
4	mm	44.4
2	mm	20.3
1	mm	13.5
0.5	mm	12.5
0.25	mm	12.2
0.125	mm	11.9
0.063	mm	11.6
**Fibres (%)**		0.3
**Binder (%)**		6.0

**Table 4:** Properties for the FE model.

Granite				Capsules		Asphalt 0.5% Capsules	
E (MPa)	55,000	υ	0.25	Yield Stress (MPa)	Plastic Strain	Yield Stress (MPa)	Plastic Strain
**Mastic**				6.56	0.00	4.77	0.00
***t_i_* (s)**	***g_i_***	***t_i_* (s)**	***g_i_***	7.96	1.55 × 10^−2^	5.38	3.13 × 10^−3^
1.00 × 10^4^	1.00 × 10^−7^	1.98 × 10^−5^	1.08 × 10^−1^	8.54	2.63 × 10^−2^	5.82	5.96 × 10^−3^
9.24 × 10^2^	5.00 × 10^−7^	1.98 × 10^−5^	1.31 × 10^−1^	8.74	3.04 × 10^−2^	6.50	1.22 × 10^−2^
7.60 × 10	1.24 × 10^−5^	1.96 × 10^−6^	2.25 × 10^−1^	8.81	3.52 × 10^−2^	6.76	1.50 × 10^−2^
8.82	1.61 × 10^−4^			9.13	4.39 × 10^−2^	7.09	1.96 × 10^−2^
1.00	1.84 × 10^−3^			9.53	5.66 × 10^−2^	7.56	2.70 × 10^−2^
9.85 × 10^−2^	1.57 × 10^−2^			9.66	7.02 × 10^−2^	7.84	3.22 × 10^−2^
1.00 × 10^−2^	7.95 × 10^−2^	***G*_0_ (MPa)**	1600	**E (MPa)**	148.00	**E (MPa)**	274.00
4.63 × 10^−4^	4.39 × 10^−1^	υ	0.30	υ	0.35	υ	0.30

**Table 5:** Compositional analysis of plant-produced asphalt.

Sieve Size		% Passing			
		M1	M2	M3	M4
14	mm	100	100	100	100
10	mm	96	95	94	96
8	mm	74	74	62	62
6.3	mm	43	48	29	29
4	mm	26	33	16	15
2	mm	17	23	12	11
1	mm	12	15	9	9
0.5	mm	10	12	8	7
0.25	mm	9	11	7	6
0.125	mm	8	9	7	6
0.063	mm	7	8	6	5
**Binder (%)**		5.7	6.9	4.8	4.1

**Table 6:** Testing of lab asphalt mixtures varying the mixing process on asphalt containing cellular capsules.

Property Measured	0% Capsules	0.5% Capsules				
		Mixing Time (%)				
		0%	20%	50%	75%	83%
Air Void content (%)	8.20	6.69	8.25	7.62	6.96	7.86
Standard deviation	0.35	0.32	0.35	0.42	0.06	0.12
Particle loss (%)	5.43	3.39	3.31	2.84	4.03	4.10
Standard deviation	0.42	0.53	0.69	0.82	0.61	0.35
Proportional rut depth (%)	4.93	4.90	4.28	3.88	3.92	4.27
Standard deviation	0.92	0.73	0.14	0.34	0.10	0.77
MPD before testing (mm)	0.96	0.89	0.91	0.90	0.88	1.02
MPD after testing (mm)	1.05	0.97	0.88	0.99	0.90	0.97
